# Metformin Regulation of the Liver Circadian Clock and Metabolic Aging: A Systems Modeling Study

**DOI:** 10.3390/metabo16030208

**Published:** 2026-03-20

**Authors:** Mengyuan Zhang, Ying Li

**Affiliations:** College of Information Technology, Shanghai Ocean University, Shanghai 201306, China; zmy_ipanda@163.com

**Keywords:** liver circadian clock, metformin, anti-aging, mathematical model

## Abstract

**Highlights:**

**What are the main findings?**

• Metformin exerts phase-dependent effects on liver circadian dynamics and metabolic aging markers.
• Feeding state critically modulates metformin responses, with fasting enhancing and high-fat diet attenuating anti-aging effects.

**What is the implication of the main finding?**

• Optimizing metformin treatment according to circadian time and metabolic state may enhance anti-aging efficacy and support more flexible drug intervention strategies.

**Abstract:**

Introduction: Aging affects both metabolic and circadian systems, leading to disruptions in energy homeostasis and phase shifts in the circadian clock. Metformin, a widely used antihyperglycemic drug, exerts anti-aging effects by modulating key pathways in the liver and also influences the liver circadian clock. However, the optimal medication strategy, including dosage and timing, that achieves significant anti-aging effects while minimizing negative impacts on the circadian clock remains unclear. This study aims to identify a rational metformin administration strategy considering both aspects. Methods: An extended mathematical model of the liver circadian clock incorporating metformin regulation was developed, and numerical simulations were performed to evaluate different dosing times and feeding conditions. Results: Metformin administration at different times produced distinct effects on both the circadian clock and anti-aging outcomes. Administration during the increasing phase of CLOCK-BMAL1 concentration showed positive effects on the circadian clock and effective anti-aging properties. Regarding feeding patterns, a fed-like state was not conducive to anti-aging, whereas fasting was beneficial. Conclusions: These findings highlight the importance of dosing time and feeding styles in optimizing metformin efficacy and provide insights into its potential pharmacological applications in anti-aging therapy.

## 1. Introduction

The physiological activities of living organisms are affected by the periodic environmental changes. To anticipate and coordinate these changes, most organisms have developed endogenous circadian clocks with a period close to 24 h. In mammals, the circadian system has two interacting parts: the core clock in the suprachiasmatic nucleus (SCN) and peripheral clocks in nearly every tissue of the body [1]. The core clock, directly synchronized with the light input, transmits rhythmic signals to peripheral clocks via hormonal pathways. Peripheral clocks play a major role in some physiological processes, such as glucose homeostasis, energy balance, blood pressure, and heart rate [2]. Among them, the liver plays a central role in maintaining energy homeostasis and responding to significant temporal changes in energy intake, storage, and utilization throughout the entire lifecycle. Compared to the systemic cues controlled by SCN, feeding and fasting cycles have a greater impact on the liver clock [3]. Aging is characterized by the gradual decline of physiological functions, which increases the risk of diseases. Many of the hallmarks of aging either affect the function of the circadian clock or are regulated by the circadian clock [4]. Previous studies have shown that aging is associated with declines in nicotinamide adenine dinucleotide (NAD+), a key metabolic cofactor in cellular redox reactions, and Sirtuin 1 (SIRT1), an NAD+-dependent deacetylase involved in metabolic regulation [5]. The changes further alter energy homeostasis by weakening the activity of AMP-activated protein kinase (AMPK), a central cellular energy sensor [6]. These main metabolic pathways interact with the circadian clock system in the liver, contributing to aging-related diseases.

Metformin is a safe, effective, and globally affordable antihyperglycemic agent that has gained much attention in recent years as a potential anti-aging treatment [7]. Previous studies in diabetic populations suggest that metformin may exert anti-aging effects beyond its glucose-lowering properties. Bannister et al. conducted a case–control study comparing T2D patients treated with metformin to non-diabetic controls and found that metformin-treated patients exhibited similar or even slightly longer lifespans than controls, despite having T2D [8]. Similarly, Ng et al. reported that in diabetic patients over 55 years old, those receiving metformin had a lower risk of cognitive decline and dementia compared to untreated patients [9]. However, the widespread use of metformin for diabetes introduces a potential confounding factor, as it remains unclear whether the observed benefits arise from improved disease management or from direct modulation of aging-related mechanisms. To address this issue, the ongoing Targeting Aging with Metformin (TAME) trial has been designed to evaluate whether metformin can delay the onset of age-related diseases in non-diabetic older adults [10].

Extensive research explored the mechanisms of metformin’s anti-aging effects, focusing on pathways such as AMPK, SIRT1, NAD+, and forkhead family of transcription factors (FOXO) [11,12,13]. Metformin can directly target presenilin enhancer 2 (PEN2) and transmit signals to the lysosomal glucose-sensing pathway through its interaction with ATPase H+ transporting accessory protein 1 (ATP6AP1), thereby activating AMPK [14]. Activated AMPK subsequently coordinates multiple downstream effects associated with anti-aging processes, including promoting FOXO phosphorylation and activating the SIRT1 pathway [15], while simultaneously inhibiting the mTOR signaling pathway [16]. In addition, metformin can directly enhance SIRT1 activity by reducing the Michaelis-Menten constant (Kₘ) for NAD+, thereby increasing the enzyme’s sensitivity to available NAD+ [12]. These coordinated modulations collectively contribute to the anti-aging properties of metformin. Regarding the crosstalk between metabolic pathways and the circadian system, AMPK has been reported to transmit energy-dependent signals to the mammalian clock by driving the phosphorylation and destabilization of CRY and PER proteins [17]. SIRT1 deacetylates the clock proteins BMAL1 and PER2 [4], thereby serving as one of the metabolic inputs to the circadian clock. The absorption, distribution, metabolism, and excretion (ADME) of metformin may exhibit time-dependent variations induced by the circadian clocks. However, it is still far from clear what impact metformin has on the circadian clock and how to use metformin to achieve significant anti-aging effects.

In addition, feeding serves as a particularly strong zeitgeber for the liver clock [3]. The anti-aging effect of metformin varies under different feeding styles [18]. Cellular metabolism in the liver is markedly affected by changes in feeding status [19]. These metabolic alterations further affect the liver circadian clock. For example, restricted feeding is known to significantly shift the circadian phase of the liver clock [20]. These findings highlight the complex connections between metformin’s pharmacological actions, metabolic regulation, and circadian rhythms, especially the impact of feeding styles on the anti-aging efficacy of metformin, which deserves further research.

Experimental studies have revealed important molecular mechanisms underlying the intricate interactions among circadian regulation, liver metabolism, and pharmacological effects. These processes involve numerous genes and signaling pathways whose interactions are highly complex. Understanding how drug administration timing influences such systems across the circadian cycle, therefore, requires integrative approaches. Mathematical modeling has been widely applied to explore the regulatory mechanisms of the circadian clock and related biological processes [21,22], providing a powerful framework to synthesize existing biological knowledge and predict system-level responses under different drug intervention strategies.

Several studies have demonstrated the potential of mathematical modeling in investigating pharmacological regulation of biological systems. Sadria and Layton developed a model to simulate the effects of drugs such as rapamycin and wortmannin on metabolic pathways and longevity by modulating key targets, including mechanistic target of rapamycin complex 1 (mTORC1) [23]. By integrating the influence of circadian rhythms on the neuroendocrine-immune system, Meyer-Hermann et al. created a mathematical model and predicted the optimal timing for glucocorticoid administration to inhibit tumor necrosis factor (TNF) [24]. In addition, Best et al. coupled a mathematical model of melatonin synthesis with a circadian clock model and investigated how melatonin dosing time influences circadian phase [25]. Previous studies have also explored the mechanisms of metformin, focusing on its effects on signaling pathways, energy metabolism, and circadian regulation [26,27,28]. Despite these advances, how metformin should be optimally administered to maintain drug efficacy while minimizing disruption to the circadian clock remains unclear. In this context, mathematical modeling can assist experimental studies by enabling hypothesis generation and guiding optimization prior to empirical validation.

In this article, we design a mathematical model of metformin action based on the existing biological mechanisms of metformin effects and the liver clock model. The anti-aging effects of different dose times and dosages, as well as their impact on the circadian clock, are studied systematically. Based on its pharmacological effects and impact on the circadian clock, it is recommended to determine the optimal strategy of metformin administration.

## 2. Materials and Methods

### 2.1. Description of the Mathematical Model

**Figure 1:**
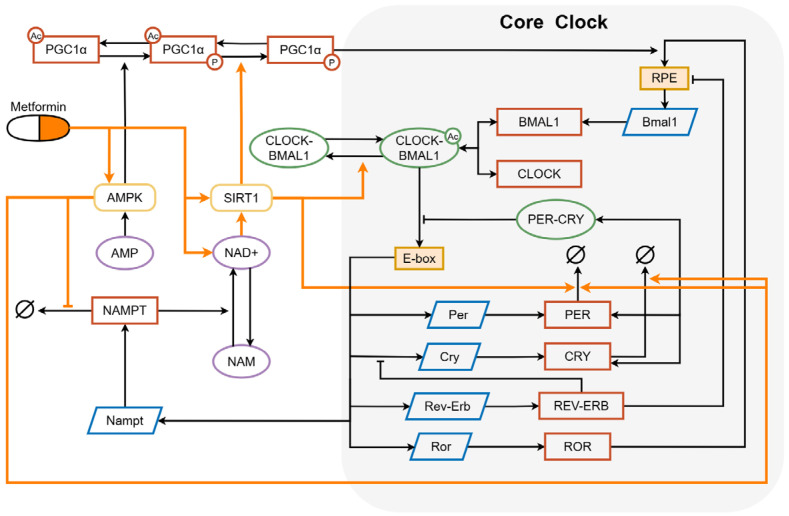
Diagram of the mathematical model describing the core clock (in the gray box) driven by the metabolism system in the liver with the regulation of metformin. The pathways related to metformin’s regulation are indicated by orange arrows. Acetylation and phosphorylation are indicated with small circles. The full meanings of all abbreviations in the figure are listed in Abbreviations.

The liver plays an important role in regulating metabolic homeostasis during the aging process. As shown in Figure 1, the liver circadian clock is tightly linked with metabolic feedback loops at the molecular level. Energy sensors such as AMPK, SIRT1 and NAD+ serve as metabolic inputs to the clock. The molecular basis of the liver circadian clock consists of a set of core clock genes that maintain oscillatory expression through a series of feedback regulations. In the positive feedback loop, CLOCK and BMAL1 proteins regulate the transcription of Per and Cry genes. Then the transcriptional products, PER and CRY proteins, translocate to the nucleus and prevent CLOCK-BMAL1 complex from binding to E-box elements, which inhibit their transcription processes in turn, thereby forming the negative feedback loop. In the auxiliary feedback loop, REV-ERB and ROR proteins bind to RRE, respectively repressing and activating the positive feedback loop.

Metformin displays its anti-aging effect by activating a network of energy sensors. AMPK activated by metformin drives SIRT1 phosphorylation, a key step, which promotes the cell autophagy process. There is evidence that metformin also activates SIRT1, possibly through the reduction in the Michaelis–Menten constant ($K_{m}$) for NAD+ [12]. Finally, activated AMPK influences the liver clock by promoting the degradation of PER and CRY proteins, while SIRT1 also plays a role in promoting the degradation of PER proteins. In addition, metformin-induced elevation of SIRT1 enhances the deacetylation rate of CLOCK-BMAL1. Woller et al.’s liver clock model not only describes the mammalian liver circadian clock but also integrates key metabolic sensors, including AMPK, NAD+, and SIRT1 [3]. Since metformin exerts its effects through metabolic pathways that interact with the liver circadian clock, Woller et al.’s model is well-suited for analyzing the effects of metformin within a unified mechanistic framework. In Woller et al.’s model, the mRNA expression profiles were obtained from mouse liver transcriptomic data reported by Hughes et al. [29], while the NAD+ and AMP measurements were derived from metabolite data reported by Hatori et al. and Ramsey et al. [30,31]. These datasets were obtained from wild-type mice under standard conditions, which allows us to investigate the effects of metformin on anti-aging without the additional complexity introduced by disease states. In this article, we extended Woller et al.’s liver clock model by incorporating the regulation of metformin. The model is formulated within a deterministic framework and consists of 16 ordinary differential equations (ODEs) together with 4 algebraic functions describing the dynamics of the circadian-metabolic network. Numerical simulations were performed in Matlab (version 2016) using standard ODE solvers. Detailed equations are provided in Appendix A.

### 2.2. Design of Metformin Regulation in the Mathematic Model

The modeling strategy of metformin is summarized as follows. First, based on experimental findings, the main biological mechanisms through which metformin exerts its effects are identified. Woller et al.’s model is then examined to determine the parameters that correspond to these biological processes. Sensitivity analysis of the SIRT1 peak value is subsequently performed to determine whether these parameters should be increased or decreased to represent the regulatory effects of metformin. Finally, a metformin pulse described by a mathematical function dynamically modulates these parameters over time, thereby providing a representation of the pharmacological action of metformin in the model.

Experimental results have demonstrated the biological mechanisms and pathways of metformin’s anti-aging effects [32]. Metformin activates AMPK, promoting the phosphorylation of SIRT1 while also influencing the synthesis of Per and Cry mRNA in the circadian system [4,17]. Additionally, metformin activates SIRT1 by reducing the Michaelis-Menten constant ($K_{m}$) of NAD+. But how to express these biological mechanisms in the mathematical model requires us to design. We conduct the sensitivity analysis of the max value of SIRT1 to parameters related to these pathways, which reflects the alterations of the SIRT1 peak value resulting from parameter perturbations in the model. Related parameters are listed in Table 1.

**Figure 2:**
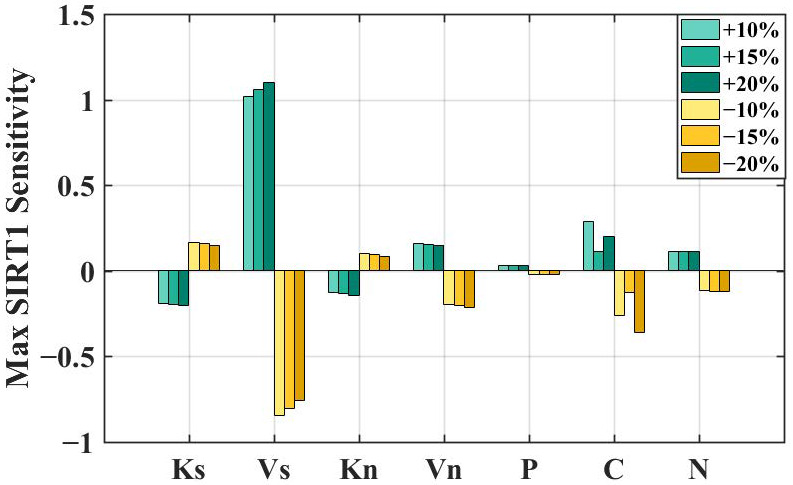
Sensitivity analysis of parameters on the peak value of SIRT1. These parameters are related to the pathways of metformin regulation. Decreasing and increasing their values result in different feedback effects on the max SIRT1 value. Abbreviations: Ks ($K_{sirt}$), Vs ($V_{sirt}$), Kn ($K_{nam}$), Vn ($V_{nad}$), P (m_per_ampk), C (m_cry_ampk), and N (m_nampt_ampk). Note: The values of S corresponding to m_per_ampk and m_cry_ampk are scaled by 1000× and 500×, respectively, for visualization.

The sensitivity of the max value of SIRT1 is described as follows [21]:

(1) $$S=\frac{\partial \ln{SIRT}_{max}}{\partial \ln p_{i}}=\frac{p_{i}}{{SIRT}_{max}}\cdot \frac{\partial {SIRT}_{max}}{\partial p_{i}},$$

where ${SIRT}_{max}$ is the max value of SIRT1, and $p_{i}$ is the parameter with index i. It is difficult to fully study the dynamic properties of the system in the whole parameter space. In order to evaluate the sensitivity to changes in the parameters, we vary one parameter value at a time and keep the rest fixed. Parameter variations of ±10%, ±15%, and ±20% were applied to assess the stability of the system in response to these metformin-related parameter changes. The results are shown in Figure 2. For each parameter, the sensitivity of the max SIRT1 maintains the same sign across the positive perturbations (+10%, +15%, and +20%) and across the negative perturbations (−10%, −15%, and −20%). This consistency indicates that the qualitative response of the system is preserved under parameter perturbations. The system behavior is robust with respect to these metformin-related parameters.

According to the results of the sensitivity analysis shown in Figure 2, we further assessed whether specific parameters should be increased or decreased to represent the regulatory effect of metformin in the model. If the max SIRT1 sensitivity becomes positive when a parameter is decreased, the parameter is reduced in the model through the metformin pulse to mimic administration. These parameters are Ks ($K_{sirt}$) and Kn ($K_{nam}$). Conversely, if the max SIRT1 sensitivity becomes positive when a parameter is increased, the parameter is increased in the model through the metformin pulse. These parameters are Vs ($V_{sirt}$), Vn ($V_{nad}$), P (m_per_ampk), C (m_cry_ampk), and N (m_nampt_ampk).

Based on the above analysis, the metformin pulse is incorporated into the model through parameter-specific formulations that represent either up-regulation or down-regulation effects:

(2) $$\;{P^{’}}_{MFD}=\frac{P_{MFD}}{1+Metformin},$$

(3) $${\;\;\;\;\;\;\;\;\;\;\;\;\;\;\;\;P^{’}}_{MFI}=P_{MFI}\cdot \left(1+Metformin\right).$$

Here, $P_{MFD}$ represents the parameters to be decreased to mimic metformin administration, including $K_{sirt}$ and $K_{nam}$. $P_{MFI}$ stands for the parameters to be increased to mimic metformin administration, including $V_{sirt}$, $V_{nad}$, m_per_ampk, m_cry_ampk, and m_nampt_ampk. ${P^{’}}_{MFD}$ and ${P^{’}}_{MFI}$ denote the corresponding parameters with metformin administration.

The metformin administration and feeding schedules are mathematically described by adjusting the Input Signal Step Function (ISSF) [3,33]. The ISSF is a periodic function with multiple parameters that can reproduce a wide variety of waveforms, thereby allowing flexible representation of both metformin administration and feeding-induced AMPK dynamics.

Metformin administration and AMPK activities are described as follows:

(4) Metformin=amp·P,

(5) Act_AMPK=(1−Campk)·offs+Campk·(amp1·P+amp2·P),

(6) $$P=P\left(t,t_{c},T_{d},\;T_{w},T_{r},24\right),$$

where amp, amp1, and amp2 represent the amplitudes of their pulses. Campk and offs are used to describe perturbations. For the details of P, please see Appendix A or refer to Ref. [3].

**Figure 3:**
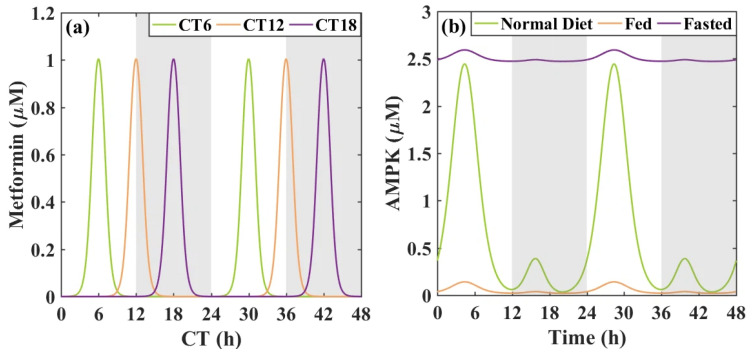
Curves of external inputs used in the model. (**a**) Pulses are conducted for metformin administered at CT6, CT12, and CT18; (**b**) AMPK activity curves describe different feeding schedules. AMPK levels for a fed-like state (constantly low AMPK activity), a fasted-like state (constantly high AMPK activity), and a normal diet state [3]. The background shading (white and gray) indicates the subjective day and night phases, respectively, and is included for visual reference only.

Figure 3a shows the metformin administration at different circadian times (CTs). CT denotes circadian time, a relative time scale commonly used to represent phases of the circadian cycle. AMPK activity curves describing different feeding schedules (normal diet, fed, and fasted) are shown in Figure 3b. Normal diet corresponds to the baseline AMPK activity, representing a typical metabolic state. The fed state corresponds to low AMPK activity, reflecting the suppression of AMPK due to sufficient energy availability after high-fat feeding. The fasted state corresponds to high AMPK activity, as cells activate AMPK to maintain energy balance during food deprivation.

To study the anti-aging effect of metformin and its impact on the liver circadian clock, we simulate the process of aging by lowering the values of parameters related to SIRT1 and NAD+ [34]. In this study, aging is described as a relative physiological state compared with the young control condition rather than a specific chronological age. Notably, we reduce the maximum SIRT1 activity ($V_{sirt}$) to simulate the decline of SIRT1 activity observed in aging people. Additionally, among the parameters related to NAD+, we lower the threshold for NAD+ to NAM conversion inactivation (NAD_basal) and the total concentration of NAD+ and NAM (NAD_tot) to reflect the reduced NAD+ levels during aging.

## 3. Results

### 3.1. Effects of Aging on Metabolism and the Circadian Clock

Aging is a multifactorial process characterized by gradual declines in physiological functions. In the liver, such declines are mainly manifested in both metabolism and the circadian clock. SIRT1 and NAD+ play a crucial role in translating the regulation of energy metabolism in the aging process.

**Figure 4:**
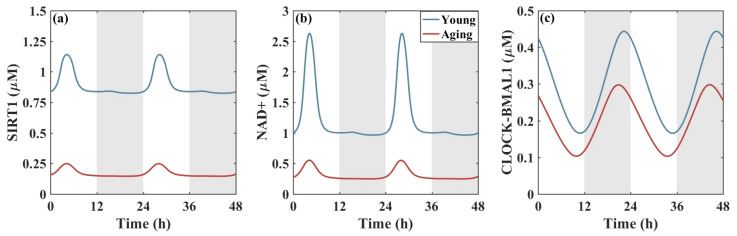
Effects of aging on metabolism and the liver circadian clock. The aging-induced decline in SIRT1 (**a**) and NAD+ (**b**) results in reduced CLOCK-BMAL1 levels (**c**) and a shortened circadian period. The background shading (white and gray) indicates the subjective day and night phases, respectively, and is included for visual reference only.

The effects of aging on metabolism and the circadian clock are shown in Figure 4. Numerical simulations of the mathematical model were performed in Matlab (version 2016), and the stable oscillatory solutions of SIRT1, NAD+, and CLOCK-BMAL1 were obtained. From Figure 4a,b, we find that aging lowers SIRT1 and NAD+ levels. Here, we use the key clock protein CLOCK-BMAL1 as a representative of circadian rhythms. We find that aging reduces the amplitude and period of protein CLOCK-BMAL1, as shown in Figure 4c. The endogenous period of the circadian clock is reduced from 24.2 h to 22.3 h with aging. This reduced amplitude is consistent with the fact that aging gradually weakens rhythmicity. The shortened period in elderly people is also consistent with their daily rhythm of going to bed early and getting up early.

In the liver, metabolism and the circadian clock are tightly integrated. The NAD+-dependent deacetylase SIRT1 and the AMP sensor AMPK directly target core clock genes [35]. SIRT1 enhances Bmal1 expression by promoting the rate of deacetylation of peroxisome proliferator-activated receptor-γ coactivator 1-α (PGC1-α). Therefore, reduced SIRT1 leads to decreased CLOCK-BMAL1 levels.

### 3.2. Influences of Metformin Administration on the Circadian Clock

Treatment with metformin might cause impairment of circadian clocks, especially if given at an inappropriate time [36]. The impact of metformin administration on the liver circadian clock should be taken into consideration. Phase is an important indicator closely related to the entrainment of the circadian rhythm with the external environment. It is necessary to examine the impact of metformin on the circadian clock from a phase perspective. It is an effective method to draw phase response curves (PRCs) for studying the phase shifts induced by external signals added at different CTs. In addition to phase, amplitude also plays an important role in the circadian clock, which is related to the strength of the circadian rhythm. For mammals, it has been found that patients with schizophrenia, depression, or cancer do not have a circadian clock or have a very weak circadian clock (low amplitude). Similarly, making amplitude response curves (ARCs) is also an effective way to study the amplitude changes induced by external signals added at different CTs.

**Figure 5:**
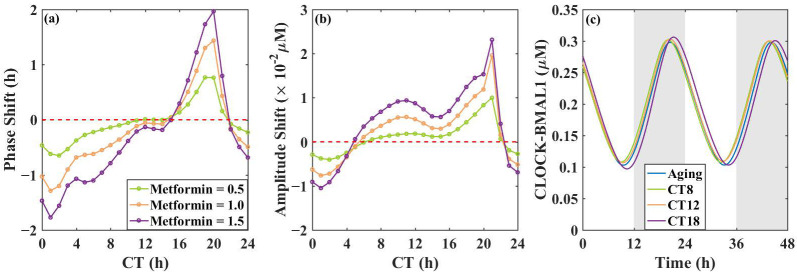
Influences of metformin administration on the liver circadian clock. (**a**) PRCs of the circadian clock with different amounts of metformin; (**b**) ARCs of the circadian clock with different amounts of metformin; (**c**) the time series of CLOCK-BMAL1 under three dosing times (CT8, CT12, and CT18) at a metformin amount of 1.0 µM. Positive values indicate phase delay, while negative values indicate phase advance in (**a**). Positive values indicate the increase in amplitude, while negative values indicate the decrease in amplitude in (**b**). Metformin is added to the aging model. The background shading (white and gray) indicates the subjective day and night phases, respectively, and is included for visual reference only.

To see the impacts of metformin administration on the phase and amplitude more clearly, we draw the PRCs and ARCs of CLOCK-BMAL1 after one day of metformin dosing, as shown in Figure 5. In this study, CT0 is defined as the time when CLOCK-BMAL1 reaches its peak in the aging group, providing a relative circadian time reference rather than an absolute experimental time. Metformin was administered at 25 circadian time points from CT0 to CT24. The phase difference and amplitude difference in CLOCK–BMAL1 before and after treatment were computed to construct the PRCs and ARCs.

The result shows that administering metformin at different CTs has varying influences on the phase shift and amplitude of the liver circadian clock. As shown in Figure 5a, metformin administration between CT = 0 h and CT = 10 h advances the circadian clock, while metformin administration between CT = 16 h and CT = 21 h delays the circadian clock. There is a zone between CT = 11 h and CT = 15 h where the phase shifts are approaching zero, which means melatonin administration has little effect on the circadian phase. Figure 5b shows that the impact on amplitude is relatively small. Although it increases the amplitude at most of the CTs, the increase is very slight. Additionally, the effect is proportional to metformin dosage—the greater the dosage, the larger the phase shift and the more significant the amplitude increase.

From Figure 4, we know that aging shortens the circadian clock period and advances the phase. Therefore, administering metformin between CT16 h and CT21 h can mitigate the phase advancement caused by aging, thereby alleviating early morning awakenings in the elderly. In addition, administering metformin enhances rhythm levels to a certain extent, although the effect is not significant. From a rhythmic perspective, administering metformin between CT16 and CT21 is optimal, as it can both delay the phase and enhance the amplitude. In order to see the impact of metformin at different CTs more intuitively, we select three representative time points of 1.0 µM metformin administration to display the time evolution diagram of CLOCK-BMAL1 in Figure 5c.

Our observation that the effect of metformin administration on phase is different when administered at different CTs aligns with Henriksson et al.’s report that metformin activates AMPK in mice differently at different times, and this phenomenon depends on the functional circadian clock [37]. This consistency suggests that dosing-time-dependent phase shifts in the circadian clock may underlie temporal variations in metformin’s pharmacological efficacy.

### 3.3. Effects of Metformin Administration on Anti-Aging

**Figure 6:**
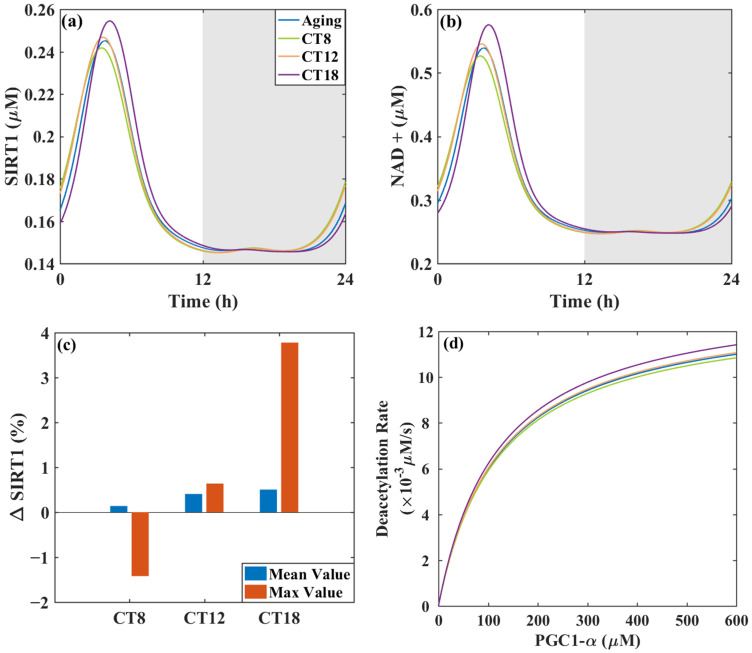
Effect of dosing schedules on metformin efficacy. (**a**,**b**) The time series of SIRT1 and NAD+ under different dosing schedules. (**c**) The percentage changes in SIRT1’s mean and max value after metformin administration. (**d**) The deacetylation rates of PGC-1α show a slight difference among different dosing times. The background shading (white and gray) indicates the subjective day and night phases, respectively, and is included for visual reference only.

Based on the impact of metformin administration on the circadian clock, we select three typical medication administration time points, CT8, CT12, and CT18, which respectively advance the phase, barely change the phase, and lag the phase, to study the anti-aging effect of metformin administration. We change parameters in the mathematical model that describe the metformin regulatory pathways to mimic metformin dosing. The pharmacodynamics of metformin differ markedly among these three dosing times, as Figure 6 depicts.

As previous work demonstrates that SIRT1 influences apoptosis, circadian rhythms, and metabolism [38], we assess metformin’s anti-aging effect by measuring the variations in the mean and maximum SIRT1 levels compared with the controlled aging model, calculated following 24 h of metformin administration, when SIRT1 and NAD+ in the model have re-attained steady state (see Figure 6a,b). The percentage changes in the mean and maximum SIRT1 values were calculated by comparing the SIRT1 data at CT8, CT12, and CT18 over a 24 h cycle with those of the aging group. As shown in Figure 6c, metformin administration at CT12 and CT18 induces an increase in max SIRT1 value of 0.66% and 3.78%, respectively. However, metformin administration at CT8 leads to a decrease in the max SIRT1 value of 1.42%, which is not a positive impact. In terms of max value, its increases may be mostly influenced by the transient dosing pulse. For anti-aging molecular mechanisms, such transient enhancement may support the physiological processes that activate above a SIRT1 threshold. Metformin administration at these three times all increases the mean value of SIRT1 to varying degrees, which indicates an enhancement of its overall activity following drug treatment. Among them, CT8 (or CT18) shows the least (or largest) increase in the mean value of SIRT1. This suggests that the drug exerts a sustained activation effect on SIRT1 after a metformin pulse influence. The elevation in the mean SIRT1 level represents the sustained nature of such effects. Overall, CT18 is the best choice from both the maximum and mean perspectives. And time points like CT8 are medication administration times that should be avoided.

Anti-aging drugs such as resveratrol activate SIRT1, leading to the deacetylation of PGC-1α, which mechanistically mitigates age-related mitochondrial functional decline [23]. Higher SIRT1 levels lead to an increased deacetylation rate of PGC-1α. The anti-aging effects of metformin at different dosing times were also assessed by computing the PGC-1α deacetylation rate based on Michaelis-Menten kinetics [39]. At baseline PGC-1α $K_{m}$ level, metformin enhances the PGC-1α deacetylation rate, with a better effect observed at CT18 than at CT12, while CT8 shows a negative effect compared to the aging group, as shown in Figure 6d. This indicates that metformin administration at CT18 activates more SIRT1 to participate in the deacetylation of PGC-1α, thereby exerting the best anti-aging effect among the three administration time points.

**Figure 7:**
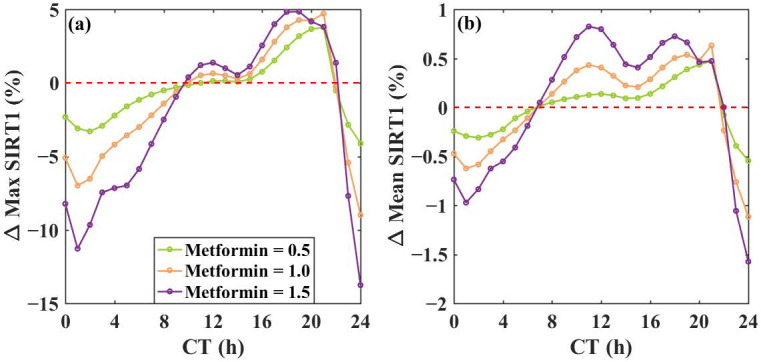
Efficacy of metformin at different doses and CTs. (**a**) The change in the maximum value of SIRT1 with different amounts of metformin. (**b**) The change in the mean value of SIRT1 with different amounts of metformin. Positive values represent an increase. Metformin is added to the aging model.

In order to comprehensively evaluate the anti-aging effects of metformin administered at different times and dosages, we simulated the model and calculated the peak and mean SIRT1 levels for each CT. The percentage changes in max and mean SIRT1 were then computed relative to the aging group (no metformin). Response curves of these changes across different CTs were plotted to visualize the time-dependent effects of metformin administration (see Figure 7). For the max SIRT1 value, metformin administration at CT0–CT6 induces a marked decrease, whereas dosing at CT15–CT21 enhances the SIRT1 peak, with the stronger effect observed at higher doses. In terms of the mean SIRT1 value, metformin administration at CT8–CT21 induces a positive shift, whereas dosing at CT0–CT7. Because the endogenous period of the aging model is about 22 h, the effects of metformin administration at CT23 and CT24 are the same as those of CT1 and CT2. Based on the results shown in Figure 7, it can be concluded that CT8 to CT21 is an optimal period for metformin administration. Particularly, CT15 to CT21, as administering metformin during this period yields the greatest increase in both the max and mean values of SIRT1.

### 3.4. Different Feeding Schedules Influence Metformin’s Anti-Aging Effect

**Figure 8:**
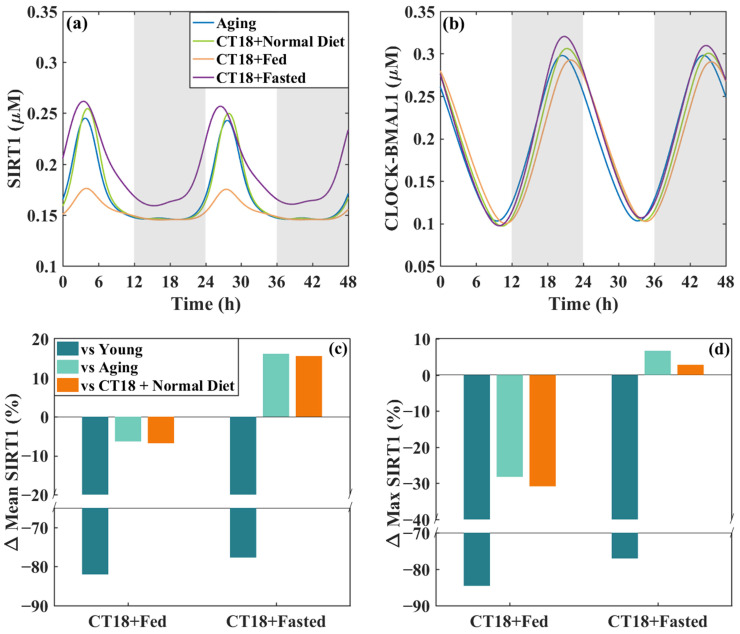
Influences of feeding schedules on metformin’s anti-aging effects. (**a**,**b**) The time series of SIRT1 and CLOCK-BMAL1. CT18+Normal Diet, CT18+Fed, and CT18+Fasted stand for adding metformin at CT18 with the Normal Diet, the Fed, and the Fasted on the aging control model, respectively. (**c**,**d**) Quantitative comparison of mean and max SIRT1 levels. Vs Young, vs aging, and vs CT18+Normal Diet mean the differences between CT18+Fed (or CT18+Fasted) and the young model, aging model, and adding metformin at CT18 with the Normal Diet on the aging model, respectively The background shading (white and gray) indicates the subjective day and night phases, respectively, and is included for visual reference only.

AMPK is a key energy sensor in biological metabolism. It is noted that AMP levels are reduced in the livers of mice fed a high-fat diet, leading to decreased AMPK activity [30]. Here, we examine the influence of feeding schedules on the anti-aging effects of metformin dosing at CT18. From Figure 5 and Figure 6, one knows that dosing at CT18 brings phase delay to the liver circadian clock, which counteracts age-related phase advancement. It also yields the largest increases in both the maximum and mean values of SIRT1 compared with CT8 and CT12. That is to say, adding metformin at CT18 can achieve the best anti-aging effect and also have a positive regulatory effect on the circadian clock. Therefore, we chose to add metformin at CT18 as our research object. The results are shown in Figure 8.

Normal diet corresponds to a typical metabolic state, the fed case corresponds to high-fat feeding, and the fasted case corresponds to food deprivation. Figure 8a,b display the time series of SIRT1 and CLOCK-BMAL1 under different feeding conditions. Compared to the aging case, adding metformin at CT18 to the normal diet has a slight anti-aging effect, and the fasted feeding amplifies the anti-aging effect of metformin. However, the fed case greatly weakens the anti-aging properties of metformin, as shown in Figure 8a. From the perspective of its impact on the circadian clock, adding metformin at CT18 with the normal diet causes a phase lag and an increase in amplitude, thereby improving the rhythmic level and delaying the phase of the circadian clock. The fasted case is conducive to the positive regulation, and the fed case counts against the regulation of metformin as it decreases the amplitude of CLOCK-BMAL1, as shown in Figure 8b. The percentage changes shown in Figure 8c,d were obtained by comparing the SIRT1 levels in the CT18+Fed and CT18+Fasted conditions with three reference groups: the young model, the aging model, and the aging model treated with metformin at CT18 under normal diet. Figure 8c,d show that the fed case reduces both the maximum and mean values of SIRT1, while the fasted case increases them. This further illustrates that the fed weakens the anti-aging properties of metformin, while the fasted contributes to it. However, regardless of diet and medication, the level of SIRT1 cannot return to a youthful state. That is to say, medication and diet can only delay or slow the aging process, but cannot fully return to a youthful state.

## 4. Discussion

Aging is a multifactorial biological process involving chronic diseases that manifest from the molecular level to the systemic level [40]. Studies indicate that aging mice exhibit reduced neurotransmitter signaling in the SCN [4], which causes the decreased phase synchronization with other peripheral clocks [1]. In addition, it has been indicated that circadian desynchrony is linked to metabolic diseases [41]. Moreover, research has demonstrated that aging is associated with reductions in both SIRT1 and NAD+ levels, which also influence the circadian clock [42]. These findings suggest that aging, metabolism, and the circadian clock are intricately interconnected. Therefore, understanding how to regulate circadian rhythms and metabolic pathways to mitigate aging has attracted increasing attention.

Metformin, which is a widely used drug in T2DM treatment, has been proven to have outstanding anti-aging ability [32]. However, the anti-aging ability of drugs is currently only in the stage of biological discovery, and as far as we know, there has not been a systematic study on their kinetics. Especially how taking metformin can produce the best anti-aging properties is still far from clear. As feeding is a particularly strong zeitgeber for the liver circadian clock, the anti-aging effects of metformin may differ depending on the feeding schedule. Additionally, metformin administration also has an impact on the liver circadian clock, which may cause impairment of circadian clocks, especially if given at an inappropriate time [36].

To address these issues, we developed an extended mathematical model based on the model proposed by Woller et al. [3]. Specifically, based on the known mechanisms of metformin, its effects are incorporated into the model by adjusting a set of key parameters that represent the corresponding regulatory pathways in the system. Using this model, we systematically investigated the effects of metformin administration at different circadian times (CTs) on circadian rhythmicity and anti-aging efficacy. In this study, CT0 is defined as the time when CLOCK-BMAL1 reaches its peak.

The effect of metformin on the circadian clock is studied from both amplitude and phase perspectives. The amplitude of CLOCK-BMAL1 reflects circadian rhythmicity, while phase indicates circadian stability. Simulation results indicate that metformin generally enhances rhythmicity at most times, except near the CLOCK-BMAL1 peak (CT0–CT5, CT23–CT24), though the amplitude change is small. PER-CRY complexes can inhibit CLOCK-BMAL1 activity [4]. We speculate that during this period, as the CLOCK-BMAL1 peak begins to decline, the negative feedback from PER-CRY predominates, such that the metformin-induced enhancement of CLOCK-BMAL1 deacetylation by SIRT1 is effectively masked and cannot further elevate the peak level. In terms of phase regulation, metformin administration during the declining period of CLOCK-BMAL1 (CT0–CT10) tends to advance the circadian phase, whereas administration during the rising period (CT16–CT21) tends to delay it.

The anti-aging efficacy of metformin was evaluated by comparing the maximum and mean levels of SIRT1 before and after drug administration. The results suggest that metformin administration during the rising period of CLOCK-BMAL1 (CT15–CT21) produces a stronger enhancement of SIRT1 levels, whereas administration during the declining period of CLOCK-BMAL1 (CT0–CT6) may lead to weaker or even negative effects. This highlights the importance of circadian timing in determining drug efficacy, as circadian clocks have been shown to modulate the biological responses to metformin [37].

Taken together, metformin administration during the CLOCK-BMAL1 rising phase (CT16–CT21) not only delays the circadian phase, thereby counteracting the age-related phase advance, but also produces the best anti-aging effect, as reflected by the largest increases in both the max and mean levels of SIRT1. This enhanced effect likely arises from the alignment of drug action with the intrinsic transcriptional activity of the circadian clock, making SIRT1 more responsive. Moreover, within the tested dose range (0.5–1.5), higher doses of metformin produce stronger effects.

Considering the impact of feeding schedules on the liver circadian clock, we further investigate the effects of metformin under different dietary conditions. The results indicate that metformin administration in the fed state weakens its anti-aging effect, whereas the fasted state enhances this effect. Medication and diet can only alleviate aging but cannot fully restore it to a youthful state.

Metabolic aging is a progressive process characterized by distinct phenotypes across different life stages [43]. The prevalence of metabolic disruptions, such as insulin resistance and metabolic syndrome, increases with age and becomes particularly pronounced in middle-aged populations [44,45]. These physiological conditions and age-related differences may significantly modulate individual responses to metformin. Future studies will therefore consider extending the current framework to incorporate different age stages and metabolic disruptions, allowing the model to explore drug effects more systematically. In addition, a study has proposed the combined administration of metformin and melatonin, a representative circadian rhythm-regulating drug, to mitigate the risk of circadian disruption induced by metformin treatment in male rats [46]. Based on the current modeling approach, we also plan to include the effects of combined melatonin administration, which may provide mechanistic insights into optimizing anti-aging interventions through coordinated circadian and metabolic regulation.

This study has several limitations. Our findings are derived from mathematical modeling and numerical simulations rather than clinical trials. As with any theoretical framework, the model simplifies complex physiological processes and focuses on specific molecular and metabolic pathways, which may not capture all individual variability. Therefore, the results should be interpreted as theoretical insights that may help guide future experimental and clinical investigations. Further experimental validation is required to confirm the predicted optimal dosing strategies and their anti-aging effects.

## 5. Conclusions

Optimizing metformin treatment according to circadian time and metabolic state may enhance anti-aging efficacy and support more flexible drug treatment strategies. Specifically, administering metformin during the CLOCK-BMAL1 rising phase (CT16–CT21) maximizes its anti-aging effect by increasing both peak and mean SIRT1 levels and counteracting age-related circadian phase advance. Feeding state further modulates the response, with administration in the fasted state enhancing the anti-aging effect, whereas administration in the fed state attenuates it.

## Figures and Tables

**Table 1:** Parameters affected by metformin treatment.

**Parameters**	**Description**
$K_{sirt}$	Michaelis–Menten constant in the NAD+-dependent SIRT1 synthesis process
$V_{sirt}$	Maximum activity of SIRT1
$K_{nam}$	Michaelis–Menten constant in the NAD+-dependent NAM synthesis process
$V_{nad}$	Maximum regeneration rate of NAD+
m_per_ampk	Modulation of Per Protein stability by AMPK
m_cry_ampk	Modulation of CRY Protein stability by AMPK
m_nampt_ampk	Modulation of NAMPT stability by AMPK

## Data Availability

The data presented in this study are available on request from the corresponding author. The data are not publicly available due to privacy concerns.

## Abbreviations

The following abbreviations are used in this manuscript:

AMPK	AMP-activated protein kinase
AMP	Adenosine monophosphate
ATP6AP1	ATPase H+ transporting accessory protein 1
Bmal1	Brain and muscle ARNT-Like 1 (gene)
BMAL1	Brain and muscle ARNT-Like 1 (protein)
CLOCK	Circadian Locomotor Output Cycles Kaput
CLOCK-BMAL1	CLOCK and BMAL1 heterodimer
Cry	Cryptochrome (gene)
CRY	Cryptochrome (protein)
CT	Circadian time
E-box	Enhancer box
mTORC1	Mechanistic target of rapamycin complex 1
NAD+	Nicotinamide adenine dinucleotide
NAM	Nicotinamide
Nampt	Nicotinamide phosphoribosyltransferase (gene)
NAMPT	Nicotinamide phosphoribosyltransferase (protein)
ODE	Ordinary differential equations
Per	Period (gene)
PER	Period (protein)
PER-CRY	PER and CRY heterodimer
PEN2	Presenilin enhancer 2
PGC1-α	Peroxisome proliferator-activated receptor gamma coactivator 1-alpha
Rev-Erb	Nuclear receptor subfamily 1, group D (gene)
REV-ERB	Nuclear receptor subfamily 1, group D (protein)
Ror	Retinoic acid receptor-related orphan receptor (gene)
ROR	Retinoic acid receptor-related orphan receptor (protein)
RPE	Retinal pigment epithelium
SIRT1	Sirtuin 1
TNF	Tumor necrosis factor
T2DM	Type 2 diabetes mellitus

## Appendix A

(a) Core clock mRNAs:

(A1)
$$\begin{array}{ccc} \frac{d[\mathrm{per}]}{dt} & = & -dm\_\mathrm{per}\cdot [per]+\left(\frac{\mathrm{Vmax}\_\mathrm{per}\cdot \left(1+\mathrm{fold}\_\mathrm{per}\cdot {\left(\frac{\left[\mathrm{CB}\right]}{\mathrm{Ka}\_\mathrm{per}\_\mathrm{cb}\cdot (1+\mathrm{Act}\_\mathrm{SIRT})}\right)}^{\mathrm{hill}\_\mathrm{per}\_\mathrm{cb}}\right)}{1+{\left(\frac{\left[\mathrm{CB}\right]}{\mathrm{Ka}\_\mathrm{per}\_\mathrm{cb}\cdot (1+\mathrm{Act}\_\mathrm{SIRT})}\right)}^{\mathrm{hill}\_\mathrm{per}\_\mathrm{cb}}\cdot \left(1+{\left(\frac{\left[\mathrm{PC}\right]}{\mathrm{Ki}\_\mathrm{per}\_\mathrm{pc}}\right)}^{\mathrm{hill}\_\mathrm{per}\_\mathrm{pc}}\right)}\right) \end{array}$$

(A2)
$$\begin{array}{ccc} \frac{d[\mathrm{cry}]}{dt} & = & -dm\_\mathrm{cry}\cdot [\mathrm{cry}]\\ & & +\left(\frac{\mathrm{Vmax}\_\mathrm{cry}\cdot \left(1+\mathrm{fold}\_\mathrm{cry}\cdot {\left(\frac{\left[\mathrm{CB}\right]}{\mathrm{Ka}\_\mathrm{cry}\_\mathrm{cb}\cdot (1+\mathrm{Act}\_\mathrm{SIRT})}\right)}^{\mathrm{hill}\_\mathrm{cry}\_\mathrm{cb}}\right)}{1+{\left(\frac{\left[\mathrm{CB}\right]}{\mathrm{Ka}\_\mathrm{cry}\_\mathrm{cb}\cdot (1+\mathrm{Act}\_\mathrm{SIRT})}\right)}^{\mathrm{hill}\_\mathrm{cry}\_\mathrm{cb}}\cdot \left(1+{\left(\frac{\left[\mathrm{PC}\right]}{\mathrm{Ki}\_\mathrm{cry}\_\mathrm{pc}}\right)}^{\mathrm{hill}\_\mathrm{cry}\_\mathrm{pc}}\right)}\cdot \frac{1}{1+{\left(\frac{\left[\mathrm{Prot}\_\mathrm{rev}\right]}{\mathrm{Ki}\_\mathrm{cry}\_\mathrm{rev}}\right)}^{\mathrm{hill}\_\mathrm{cry}\_\mathrm{rev}}}\right) \end{array}$$

(A3)
$$\begin{array}{ccc} \frac{d[rev]}{dt} & = & -dm\_rev\cdot [rev]+\left(\frac{Vmax\_rev\cdot \left(1+\mathrm{fold}\_\mathrm{rev}\cdot {\left(\frac{\left[CB\right]}{\mathrm{Ka}\_\mathrm{rev}\_\mathrm{cb}(1+\mathrm{Act}\_\mathrm{SIRT})}\right)}^{\mathrm{hill}\_\mathrm{rev}\_\mathrm{cb}}\right)}{1+{\left(\frac{\left[CB\right]}{\mathrm{Ka}\_\mathrm{rev}\_\mathrm{cb}\cdot (1+\mathrm{Act}\_\mathrm{SIRT})}\right)}^{\mathrm{hill}\_\mathrm{rev}\_\mathrm{cb}}\cdot (1+\left(\frac{\left[PC\right]}{{\mathrm{Ki}\_\mathrm{rev}\_\mathrm{pc}}^{\mathrm{hill}\_\mathrm{rev}\_\mathrm{pc}}}\right)}\right) \end{array}$$

(A4)
$$\begin{array}{ccc} \frac{d[\mathrm{ror}]}{dt} & = & -dm\_\mathrm{ror}\cdot [\mathrm{ror}]+\left(\frac{\mathrm{Vmax}\_\mathrm{ror}\cdot \left(1+\mathrm{fold}\_\mathrm{ror}\cdot {\left(\frac{\left[CB\right]}{\mathrm{Ka}\_\mathrm{ror}\_\mathrm{cb}\cdot (1+\mathrm{Act}\_\mathrm{SIRT})}\right)}^{\mathrm{hill}\_\mathrm{ror}\_\mathrm{cb}}\right)}{1+{\left(\frac{\left[CB\right]}{\mathrm{Ka}\_\mathrm{ror}\_\mathrm{cb}\cdot (1+\mathrm{Act}\_\mathrm{SIRT})}\right)}^{\mathrm{hill}\_\mathrm{ror}\_\mathrm{cb}}\cdot \left(1+{\left(\frac{\left[PC\right]}{\mathrm{Ki}\_\mathrm{ror}\_\mathrm{pc}}\right)}^{\mathrm{hill}\_\mathrm{ror}\_\mathrm{pc}}\right)}\right) \end{array}$$

(A5)
$$\begin{array}{ccc} \frac{d[\mathrm{bmal}]}{dt} & = & -\mathrm{dm}\_\mathrm{bmal}\cdot [\mathrm{bmal}]\\ & & +\left(\frac{\mathrm{Vmax}\_\mathrm{bmal}\cdot \left(1+\mathrm{fold}\_\mathrm{bmal}\cdot (1+\mathrm{Act}\_\mathrm{PGC}1a)\cdot {\left(\frac{\left[\mathrm{Prot}\_\mathrm{ror}\right]}{\mathrm{Ka}\_\mathrm{bmal}\_\mathrm{ror}}\right)}^{\mathrm{hill}\_\mathrm{bmal}\_\mathrm{ror}}\right)}{1+{\left(\frac{\left[\mathrm{Prot}\_\mathrm{rev}\right]}{\mathrm{Ki}\_\mathrm{bmal}\_\mathrm{rev}}\right)}^{\mathrm{hill}\_\mathrm{bmal}\_\mathrm{rev}}+{\left(\frac{\left[\mathrm{Prot}\_\mathrm{ror}\right]}{\mathrm{Ka}\_\mathrm{bmal}\_\mathrm{ror}}\right)}^{\mathrm{hill}\_\mathrm{bmal}\_\mathrm{ror}}}\right) \end{array}$$

(b) Proteins of the clock:

(A6)
$$\begin{array}{ccc} \frac{d[\mathrm{Prot}\_\mathrm{per}]}{dt} & = & +kp\_per\cdot [per]\\ & & -\left(\mathrm{kass}\_\mathrm{pc}\cdot [\mathrm{Prot}\_\mathrm{cry}]\cdot [\mathrm{Prot}\_\mathrm{per}]-kdiss\_pc\cdot [PC]\right)\cdot [\mathrm{Prot}\_\mathrm{per}]\\ & & -\mathrm{dp}\_\mathrm{per}\cdot \left(1+m\_\mathrm{per}\_\mathrm{sirt}\cdot \mathrm{Act}\_\mathrm{SIRT}+m\_\mathrm{per}\_\mathrm{ampk}\cdot \mathrm{Act}\_\mathrm{AMPK}\right) \end{array}$$

(A7)
$$\begin{array}{ccc} \frac{d[\mathrm{Prot}\_\mathrm{cry}]}{dt} & = & -dp\_cry\cdot \left(1+m\_cry\_ampk\cdot Act\_AMPK\right)\cdot [\mathrm{Prot}\_\mathrm{cry}]\\ & & +kp\_cry\cdot [cry]\\ & & -\left(\mathrm{kass}\_\mathrm{pc}\cdot [\mathrm{Prot}\_\mathrm{cry}]\cdot [\mathrm{Prot}\_\mathrm{per}]-kdiss\_pc\cdot [PC]\right) \end{array}$$

(A8)
$$\begin{array}{ccc} \frac{d[\mathrm{Prot}\_\mathrm{rev}]}{dt} & = & -dp\_rev\cdot [\mathrm{Prot}\_\mathrm{rev}]+kp\_rev\cdot [rev] \end{array}$$

(A9)
$$\begin{array}{ccc} \frac{d[\mathrm{Prot}\_\mathrm{ror}]}{dt} & = & -dp\_ror\cdot [\mathrm{Prot}\_\mathrm{ror}]+kp\_ror\cdot [ror] \end{array}$$

(A10)
$$\begin{array}{ccc} \frac{d[\mathrm{Prot}\_\mathrm{bmal}]}{dt} & = & -dp\_bmal\cdot [\mathrm{Prot}\_\mathrm{bmal}]+\left(kp\_bmal\cdot [bmal]\right)\\ & & -\left(\mathrm{kass}\_\mathrm{cb}\cdot [\mathrm{Prot}\_\mathrm{bmal}]-kdiss\_cb\cdot [CB]\right) \end{array}$$

(A11)
$$\begin{array}{ccc} \frac{d[PC]}{dt} & = & +\left(\mathrm{kass}\_\mathrm{pc}\cdot [\mathrm{Prot}\_\mathrm{cry}]\cdot [\mathrm{Prot}\_\mathrm{per}]-kdiss\_pc\cdot [PC]\right)-d\_pc\cdot [PC] \end{array}$$

(A12)
$$\begin{array}{ccc} \frac{d[CB]}{dt} & = & +\left(\mathrm{kass}\_\mathrm{cb}\cdot [\mathrm{Prot}\_\mathrm{bmal}]-kdiss\_cb\cdot [CB]\right)-d\_cb\cdot [CB] \end{array}$$

(c) Metabolic pathways:

(A13)
$$\begin{array}{lll} \frac{d[\mathrm{nampt}]}{dt} & = & -dm\_nampt\cdot [nampt]\\ & & +\left(\frac{\mathrm{Vmax}\_\mathrm{nampt}\cdot \left(1+\mathrm{fold}\_\mathrm{nampt}\cdot {\left(\frac{\left[\mathrm{CB}\right]}{\mathrm{Ka}\_\mathrm{nampt}\_\mathrm{cb}\cdot (1+\mathrm{Act}\_\mathrm{SIRT})}\right)}^{\mathrm{hill}\_\mathrm{nampt}\_\mathrm{cb}}\right)}{1+{\left(\frac{\left[\mathrm{CB}\right]}{\mathrm{Ka}\_\mathrm{nampt}\_\mathrm{cb}\cdot (1+\mathrm{Act}\_\mathrm{SIRT})}\right)}^{\mathrm{hill}\_\mathrm{nampt}\_\mathrm{cb}}\cdot \left(1+{\left(\frac{\left[\mathrm{PC}\right]}{\mathrm{Ki}\_\mathrm{nampt}\_\mathrm{pc}}\right)}^{\mathrm{hill}\_\mathrm{nampt}\_\mathrm{pc}}\right)}\right) \end{array}$$

(A14)
$$\begin{array}{ccc} \frac{d[\mathrm{Prot}\_\mathrm{nampt}]}{dt} & = & -\frac{dp\_\mathrm{nampt}\cdot [\mathrm{Prot}\_\mathrm{nampt}]}{1+m\_\mathrm{nampt}\_\mathrm{ampk}\cdot \mathrm{Act}\_\mathrm{AMPK}}+\mathrm{kp}\_\mathrm{nampt}\cdot [\mathrm{nampt}] \end{array}$$

(A15)
$$\begin{array}{ccc} \frac{d([NAD])}{dt} & = & -\left(\frac{d\_\mathrm{nad}\cdot ([\mathrm{NAD}]-\mathrm{NAD}\_\mathrm{basal})}{\mathrm{Knad}+[\mathrm{NAD}]-\mathrm{NAD}\_\mathrm{basal}}\right)+\left(\frac{\mathrm{Vnad}\cdot [\mathrm{Prot}\_\mathrm{nampt}]\cdot (\mathrm{NAD}\_\mathrm{tot}-[\mathrm{NAD}])}{\mathrm{Knam}+\mathrm{NAD}\_\mathrm{tot}-[\mathrm{NAD}]}\right) \end{array}$$

(A16)
$$\begin{array}{ccc} \frac{d[dbp]}{dt} & = & -dm\_dbp\cdot [dbp]\\ & & +\left(\frac{Vmax\_dbp\cdot \left(1+fold\_dbp\cdot {\left(\frac{\left[CB\right]}{Ka\_dbp\_cb\cdot \left(1+Act\_SIRT\right)}\right)}^{\mathrm{hill}\_\mathrm{dbp}\_\mathrm{cb}}\right)}{1+{\left(\frac{\left[CB\right]}{Ka\_dbp\_cb\cdot \left(1+Act\_SIRT\right)}\right)}^{\mathrm{hill}\_\mathrm{dbp}\_\mathrm{cb}}\cdot \left(1+{\left(\frac{\left[PC\right]}{Ki\_dbp\_pc}\right)}^{\mathrm{hill}\_\mathrm{dbp}\_\mathrm{pc}}\right)}\right) \end{array}$$

(A17)
$$\begin{array}{ccc} Act\_SIRT & = & \frac{Csirt\cdot Vsirt\cdot [NAD]}{Ksirt+[NAD]} \end{array}$$

(A18)
$$\begin{array}{ccc} Act\_AMPK & = & Campk\cdot (amp1\cdot P(t,tc1,Td1,Tr1,24)\\ & & +amp2\cdot P(t,tc2,Td2,Tr2,24))+\left(1-Campk\right)\cdot offs \end{array}$$

(A19)
$$\begin{array}{ccc} \mathrm{Act}\_\mathrm{PGC}1a & = & \frac{Cpgc1\cdot Vpg\cdot Act\_AMPK\cdot Act\_SIRT\cdot Prot\_PGC1a}{1+\frac{Act\_\mathrm{AMPK}}{Kpg1}\cdot \left(1+\frac{Act\mathrm{SIRT}}{Kpg2}\right)} \end{array}$$

(A20)
$$\begin{array}{ccc} \mathrm{Prot}\_\mathrm{PGC}1a & = & amp3\cdot P(t,tc3,Td3,Tr3,24 \end{array})$$

The function P is used to describe the temporal evolution of a single pulse. It is composed of multiple step functions S and through different time shifts and period adjustments, it calculates the superposition of multiple time intervals. This function represents a modulated signal under different tim1e points, time intervals, and periodic conditions:

(A21) $$\begin{array}{ccc} P\left(t,tc,Td,Tr,Tc\right) & = & S\left(t,tc-\frac{Td}{2},0,Tw,Tc\right)-S\left(t,tc-\frac{Td}{2},Td,Tw,Tc\right)\\ & & +S\left(t,tc-\frac{Td}{2},Tc,Tw,Tc\right)-S\left(t,tc-\frac{Td}{2},Tc+Td,Tw,Tc\right) \end{array},$$

S is the step function:

(A22) $$\begin{array}{ccc} S\left(t,tf,ts,Tw,Tc\right) & = & \frac{1}{2}\left(1+tanh(\frac{t-Tc\cdot \left\lfloor \frac{t}{Tc}\right\rfloor -ts}{Tw})\right) \end{array}$$

The values of the parameters can be found in Ref. [3].
